# The Dangers of Uterine Myomata

**Published:** 1898-02-26

**Authors:** 


### THE DANGERS OF UTERINE MYOMATA.

There is no doubt that the opinion is largely held that, putting on one side certain accidents which are apt to occur during pregnancy and in labour, uterine myomata are chiefly dangerous to life by way of hæmorrhage, and that this danger is one which may fairly be expected to pass away at the menopause; but a *résumé* of the modes in which these tumours imperil life, given by Mr. Bland Sutton in the *Clinical Journal*, shows that these perils take many forms, and that the menopause is by no means always the end of the trouble.

The dangers spoken of seem not to be inherent in the tumours themselves, but to depend much upon their environment. The commonest trouble caused by myomata is no doubt hæmorrhage, but this is confined to those that implicate the endometrium. The most serious hæmorrhages are those that arise in connection with tumours which have become septic. When this happens the bleeding often continues excessive between as well as at the periods, and this, no doubt, explains the almost constant bleeding associated with partially extruded polypi. Septic infection is one of the most dangerous complications which can arise in the course of these tumours, and, in fact, when a large myoma becomes septic the danger to life is imminent. But although the danger is less urgent in the case of small tumours, it is none the less real, for the septic mischief may spread and thus set up other complications, which are themselves of great moment. Thus the tubal mucous membrane may become involved, pyosalpinx may be set up, or even inflammation of the peritoneum. In relation to this it is to be noted that the pain occurring in connection with certain fibroids may be caused by distended Fallopian tubes and not directly by the tumour.

It has often been thought that uterine myomata might undergo sarcomatous degeneration, but of this Mr. Bland Sutton is somewhat sceptical. Carcinoma, however, may, and not uncommonly does, occur in the cervix of a uterus containing a myoma, and when this happens, although the tumour remains unaffected until its capsule is eroded, it then rapidly ulcerates and sloughs.

Impaction of a fibroid in the pelvis is a matter of great importance. It may occur gradually, and may produce but few symptoms until its pressure on surrounding organs attracts attention to its presence. All varieties of myoma may become impacted, and if bleeding does not occur the tumour may have filled all the available space before its presence is discovered. A point of great importance to remember is that these myomata tend to enlarge before the menstrual period, and that pressure effects occur at that time even when at other times there may be no symptoms; so that "when a woman between 35 and 45 seeks relief because she suffers from retention of urine for a few days preceding each menstrual period, it is almost a certainty that she has a myoma in her uterus."

When a subserous myoma has a slender pedicle this may get twisted, and thus attacks of acute pain may be caused. Myomata may cause intestinal obstruction and this may be produced in several ways. If the tumour possesses a long and narrow pedicle this may entangle a loop of small intestine, and lead to fatal obstruction. Then a very large myoma may rise so high, and may press with such weight on the pelvic brim as to obstruct the sigmoid flexure, and, again, as already mentioned, the tumour may become impacted in the pelvis, and thus press upon the rectum. An accident requiring special caution sometimes arises from a small submucous fibroid, inducing inversion of the uterus, and the dangers connected with pregnancy are well recognised.

